# An Approach to the Spanish Consumer’s Perception of the Sensory Quality of Environmentally Friendly Seabass

**DOI:** 10.3390/foods10112694

**Published:** 2021-11-04

**Authors:** Juan Benito Calanche Morales, Ana Tomás-Vidal, Edilson Ronny Cusiyunca Phoco, Silvia Martínez-Llorens, Pedro L. Marquina, Miguel Jover-Cerdá, Pedro Roncalés, José Antonio Beltrán

**Affiliations:** 1 Faculty of Veterinary, IA2, University of Zaragoza, 50013 Zaragoza, Spain or juan.calanche@udo.edu.ve (J.B.C.M.); pmarquin@gmail.com (P.L.M.); roncales@unizar.es (P.R.); 2 Department of Food Technology, ECAM, University of Orient, Boca del Río 6304, Nueva Esparta, Venezuela; 3 Institute de Ciència i Tecnologia Animal, Universitat Politècnica de València, 46022 València, Spain; atomasv@dca.upv.es (A.T.-V.); roni2093@hotmail.com (E.R.C.P.); silmarll@dca.upv.es (S.M.-L.); mjover@dca.upv.es (M.J.-C.)

**Keywords:** check-all-that-apply (CATA), projective mapping, rapid sensory profiling technique, consumer research, organic diets, seafood

## Abstract

Seabass is one of the leading aquaculture species in Europe. Sensory analysis is essential for new product development. This research focused on establishing and differentiating the opinion of consumers about seabass quality obtained with organic feeding. Fish were fed for 196 days with four treatments (a control diet with 30% fishmeal and three diets with different levels of fishmeal supplemented with organic vegetable ingredients: 25%, 30% and 35%). Experimental diets were compared with commercial samples from the retail industry that were considered as “adequate quality for fish”. Two sensory analyses were carried out, check-all-that-apply (CATA) to obtain feedback on consumers’ characterization towards a different type of fish evaluated and projective mapping (PM) to measure the similarity among a set of products and establish a comparison between results provided by both methods. According to the CATA results, white color, softness, meaty taste and juicy texture were considered relevant attributes, also showing a good relationship with an adequate cooked fish description. A penalty analysis confirmed that the previous characteristics were considered essential while fibrous was an undesirable attribute. The projective mapping showed a similar sensory configuration to the CATA, corroborating these findings that showed that commercial fish were placed in a position away from the rest of the treatments, and the organic diet with a higher level of fishmeal (35%) was the most distant from the control diet.

## 1. Introduction

Seabass (*Dicentrachus labrax*) is one of the leading aquaculture species in Europe, being cultured all over the Mediterranean, the Black Sea and the North-Eastern Atlantic. The demand for its white and tasty meat, considered of high commercial interest, is primarily met by aquaculture production. Nevertheless, fishing still accounts for more than 10% of total seabass production worldwide [1]. Europe is the largest producer and exporter of this species (239.7 million t live weight), chiefly: Turkey 99.97, Greece 44.28, Spain 17.65, Italy 7.03 and Croatia 5.71 million t live weight [2]. The intense competition among countries for seabass commerce and the resulting pressure on market prices require characterization and differentiation based on product quality, also taking into account environmental sustainability. Therefore, producers and manufacturers need to know how consumers perceive their products to remain competitive [2].

The current consumer of fishery products gives increasing importance to the origin and production techniques that have been carried out throughout the entire process of product raising. Organic aquaculture arises from these new consumer demands. The organic agro-food sector shows a growing trend, and it has experienced a real boom in a short time as more and more consumers tend to buy organic food, which is environmentally friendly and different from those of intensive farming or aquaculture. Contradictory to popular belief, aquaculture does not impoverish the seas; instead, it is one of the key solutions for meeting the growing demand for fish products without further exploiting the seas and oceans [3]. Indeed, the “2030 Agenda for Sustainable Development (Global Goals for Sustainable Development)” proposed by the United Nations [4] established the need to achieve sustainable management and efficient use of natural resources. It is necessary to ensure that people everywhere have the relevant information and awareness for sustainable development and lifestyles in harmony with nature.

While substantial environmental impacts related to food occur in the production phase (agriculture, food processing), households influence these impacts through their dietary choices and habits. This consequently affects the environment through food-related energy consumption and waste generation [4]. The rise in consumers’ environmental awareness has brought forward the emergence of the “ethical” consumer, as a person who recognizes the connection between consumption and its social and ecological consequences [5]. Due to the above, the knowledge and analysis of food consumption experience becomes a vital key issue [2]. However, it should not be forgotten that the pleasure and satisfaction that consumers experience when eating fish derives from its sensory properties.

Many parameters involved in the aquaculture production process have a significant role in the final product quality. Breeding techniques, harvesting methods and slaughtering practices represent the most critical areas for sensory studies’ application. For most aquaculture operations, it is crucial to test and verify the effects of diet on the growth and development of fish that have commercial importance [2]. In aquaculture, especially in the production area, there are limited numbers of investigations that have applied novel multivariate analysis techniques to assess the sensory perception for farmed fish, including seabass. In most cases, conventional hedonic methods such as “acceptance” and “affective discrimination” tests have usually been applied [2].

Sensory and consumer research has reached a significant development. In the same way, sensometrics applied to the evaluation of data derived from this research field is forging ahead at an increasing rate [6]. These aspects are gaining increasing importance because of the consumer-specific perception of sensory quality attributes and their influence on purchase decisions [2].

Sensory profiling is an essential tool in the food science field and is key for adapting products to consumers’ preferences. The importance of using consumers to extract analytical sensory information of products has been underlined. The main argument for this is that consumers differ fundamentally in their perception of a product. Therefore, the information extracted from them is more actionable than the data obtained from trained panels [7]. The individual variability regarding consumers is particularly significant in the context of consumers’ study techniques (profiling and projecting) as the perception of the stimuli is holistic, while the way this perception is expressed is projective [8].

Check-all-that-applied (CATA) is an interesting and simple methodology to obtain an insight into consumer perceptions of foods. An interesting plot may be generated from this technique, especially when a trained sensory panel is not available or when there is not enough time to train a sensory panel [9]. In this same vein, projective mapping (PM) is a rather old technique in psychology, dating at least from 1935. The primary purpose of this method is to obtain similarity measurements among products from participants who, in general, are not required to be trained assessors [10]. As the main advantage, PM provides a global judgment about products, integrating all the sensory characteristics [11]. Data from PM are simple to collect and analyze, and will, therefore, provide simple results for the interpretation of consumer perception of products in a product development process [12]. This technique can be a useful tool in combination with preference and descriptive profile data [13].

Sensory analysis is a required tool in new product development to evaluate not only the quality but also the potential commercial viability of new foods, especially those which are environmentally sustainable. In this sense, consumer behavior research about organic fish has focused mostly on understanding the effect of information provision and related decision-making processes. However, this work was a practical application that contributed to the production of organic food (seabass fillet) whose research, development and innovation process considered the sensory response of consumers as an essential element.

Thus, this research aims to establish the opinion of consumers about the quality of seabass (*D. labrax*) produced with organic feeding and with reduced fish meal inclusion rates. Furthermore, this study compared the discriminative power from the multivariate analyses used to reach this purpose. On the one hand, it considers an already recognized and widely used method such as CATA and, on the other hand, it takes into account the projective mapping as it could represent a much more convenient, simple and quick technique to know consumers’ opinion. According to the existing literature, this is the first time that two multivariate applications (CATA and PM) have been used to discriminate and compare the sensory quality of farmed seabass produced by following different farming and feeding procedures (organic vs. traditional), based on sensory attributes that were evaluated by consumers to classify them as necessary, negative or irrelevant.

## 2. Materials and Methods

### 2.1. Fish and Sampling Procedure

The sampling procedures performed in this research were the following: batch composed of 400 seabass juveniles (initial weight of 25 g) from a local fish farm (Castellón, Spain) were transported to the Fish Nutrition Laboratory of the Universitat Politècnica de València (UPV). Once received at the laboratory, fish were randomly distributed to each feeding tank and subjected to a period of acclimation. The experimental diets used in this work were processed through a cooking-extrusion process in the feed mill of the Department of Animal Sciences of the UPV. A semi-industrial extruder of the Clextral BC45 model was used for this purpose. For the production and formulation of the experimental feed, the requirements established by the organic production regulation [14] were followed, using organic vegetable raw materials (cereals and oilseeds) with the appropriate labeling and excluding from the formulation synthetic amino acids. Three experimental diets were formulated with different levels of inclusion of fishmeal in the feeds (25%, 30% and 35%) and were named as 25% ECO, 30% ECO and 35% ECO, respectively. Another diet with 30% fishmeal replacement but without organic ingredients and the addition of free amino acids was used as a control due to it being similar to conventional diets used for raising seabass.

The trial lasted 196 days (28 weeks), and 50 fish with a medium size (final weight of 350 g on average) from the initial stock were randomly sacrificed by hypothermia by placing them in buckets of water and ice. Fish were labeled, submitted to biometric parameters and dissection, and then fillets were vacuum packed and stored in the freezer at −30 °C for 30 days.

As a reference, 12 farmed fresh seabass, less than 48 h from slaughter, were bought in two local markets in Valencia city (Spain). These were produced following common procedures in open sea cages and complementarily fed with standard feeds. This sample was denominated as “commercial”, and all fish of this trial were filleted and stored in the same conditions as those of the experimental diets, including the control. Sensory analyses were carried out on 12 fish from each treatment.

### 2.2. Sensory Assessors’ Selection and Procedure

The test took place in the test room of the Department of Aquaculture (UPV), which is designed according to ISO guidelines [14]. A sample of 100 people, including 60 men and 40 women, with ages between 18 and 64 years, of different nationalities and different incomes were considered as untrained sensory assessors according to ISO 5492:2008 [15]. They were randomly chosen from members of the university community and their families. They were asked to fill in a questionnaire regarding socio-demographic data, buying habits and other information to outline the profile of the tasters (Table 1).

All of them were regular consumers of fish, however more than half (59%) did not choose to buy fresh whole seabass since many of them preferred to buy frozen fish in different formats (fillet, steak, whole, etc.). The above information was revealed during previous interviews to know whether they bought fish and if they had any allergies to fish. However, this was not a problem due to the fact that the sensory analyses were conducted on cooked fillets from defrosted seabass that represent a traditional way of consumption.

The samples presentation was made taking into account a complete block design, where each assessor evaluated all the samples to achieve a proper balance. Fish samples were blind-coded by using 3-digit numbers, randomly selected. In each treatment tested, a fish portion (15.0 +/− 2.0 g) from the loin area was cooked in sample containers (suitable for microwave cooking) with screw caps partially closed and using a microwave oven (LG Smart Inverter 1100 W, 39 L, Seoul, Korea) [16]. A moderate warming of samples took place (600 W) for 45 s then served to the assessors directly after 30 s. Water and bread without salt (neutral) were provided to the assessors for cleansing the palate between samples. After testing, they had to register their valuation on the respective forms provided for that purpose.

### 2.3. Sensometrics Assays (CATA and PM)

Two different sensory analyses were developed; in the first place, a check-all-that-apply analysis was carried out to build a preference map and obtain information from a penalty analysis. Second, as an alternative for a quick classification, the projective mapping technique was developed to establish a projective map.

#### 2.3.1. CATA Analysis

The consumers’ panel previously selected was used to carry out this analysis. According to the recommendations of previous researchers that set a minimum number of 80 people [17] and another one that recommended between 100–150 [18], the size of this panel was therefore enough for reliable sensory characterizations. However, to assess an adequate sample size used in this research a power analysis was developed using G*Power^®^ software V.3.1.9.7 (University of Kiel, Germany), and the output (α = 0.05 and 1-β = 0.951) indicated that the size used (*n* = 100) was satisfactory taking into account the total size suggested (*n* = 90) and the Power (0.95) of the test. The vocabulary of the sensory attributes was developed based on descriptors collected in the tables “Definitions of some terms used in sensory assessment of fish” and “Texture profile” proposed by Seafish [19]. A total of 62 terms generated to describe all fish species were considered. However, taking into account sensory descriptors collected in the “Torry scheme for cooked seabass” [20], an optimized list of specific sensory attributes was employed in this analysis. The CATA ballot consisted of a list of 45 terms (Table 2), comprising sensory attributes. It was divided into sensory modalities with a shortlist for each one to avoid a “dilution effect of responses” [21], for which the presentation followed the “dynamics of sensory perception” (odor, color of the meat, flavor and texture) to lower the cognitive effort required by consumers [7,22]. Before the development of the CATA, participants received a brief talk about the methodology to be used, as well as the sensory attributes to be identified, which were defined and collected in a list provided to them before. They had to read the whole document and then there was a turn to speak and to clarify any doubts or concerns. Furthermore, they were allowed to keep the attributes list during the sample evaluation process.

During the test, assessors received the assessment instrument (paper document) consisting of the CATA ballot and acceptance test (5-point hedonic scale) to assess cooked fillets from defrosted seabass.

#### 2.3.2. Projective Mapping (PM)

Fillets from experimental diets [3] and the commercial sample [1] were coded with three random digits, and the sensory assessors were asked about them. Once they had tasted all 5 samples, they had to place a sample container of each treatment inside a square drawn (110 mm × 150 mm) on the paper (A4), according to the similarities or differences found.

Assessors were told that two products should be placed very close to each other if they were perceived as identical and far from each other if they were perceived as very different [23,24]. No further instruction was provided about how the samples should have been separated in this space, and so each assessor chose his criteria [25]. For each judgment, the coordinates (X, Y) corresponding to the positioning of the sample container within the box were measured, collected and then processed.

#### 2.3.3. Comparative Study between Methods (CATA vs. PM)

The preference of consumers for tested diets and commercial fish was compared for both the sensory methods (CATA and PM) applied. The methodology proposed by Esmerino et al. (2017) [26] using a similarity study between sensory configurations was carried out. The preference of consumers for tested diets and commercial fish was compared for both the sensory methods (CATA and PM) applied. First, a hierarchical cluster analysis (HCA) was performed on the samples’ coordinates in the first and second dimensions in the space defined by multiple factorial analysis (MFA) for PM and correspondences analysis (CA) in the case of CATA to identify groups of samples with different sensory characteristics. This approach has been used with success in previous studies to evaluate and determine similarities and differences among sensory methods [23]. Then the similarity between configurational congruence [27] for both methods was matched by applying MFA on the cross-tabulation matrix for all methods studied. Finally, RV coefficients, the correlation coefficient between two individual spaces, ranging from 0 (totally disagree) to 1 (perfect agreement) was calculated, and the significance was determined using the standardized RV.

### 2.4. Statistical Analyses

Data collected using CATA questions were analyzed using standard procedures [22,28]. The frequency of use of each CATA term was determined by counting the number of consumers that used that term to describe each sample. Commercial fish were considered as “acceptable food” since these represented the quality of farmed seabass to which consumers are accustomed. Cochran’s Q test (*p* < 0.05) was carried out separately on data from each experimental treatment to identify significant differences among products for each of the sensory terms, then a multiple pairwise comparisons (MPC), using the Marascuilo procedure, was carried out. Correspondence analysis (CA) was performed on the frequency table from each experimental treatment, considering Hellinger’s distances similarity/dissimilarity tool. Then, a correlation matrix including attributes (tetrachoric correlation) and liking scores (biserial correlation) was developed. From this one, principal coordinates analysis (PCoA) was applied to the φ correlation coefficients [29], and results were visualized in a two-dimensional map according to the procedure for graphical analysis proposed by Meyners et al. (2013) [28]. Thus, proximities among attributes could be analyzed. Finally, a penalty analysis with liking scores was developed [30]. Regarding the results acquired for the projective mapping, it was analyzed by MFA to evaluate several tables of variables simultaneously, and to obtain results, particularly charts, that allowed the study of the relationship between the observations, the variables and the tables [31]. In the comparative study between methods, HCA was performed on the samples’ coordinates in the first and second dimensions in the space defined by MFA and CA to identify groups of samples with different sensory characteristics considering Euclidean distances (dissimilarity), Ward’s aggregation criterion (agglomeration method) and automatic truncation. Meanwhile, MFA was displayed in a plot where points at the end of each representation showed the position according to the sensory method. In contrast, the central point (mean) represented a consensus position between them. By visual inspection of it, samples positions for all methodologies were analyzed. The software XLSTAT 2021.3.1 (Addinsoft^®^, New York, NY, USA) was used for developing statistical analyses.

### 2.5. Ethical Statement

The experimental protocol was reviewed and approved by the Committee of Ethics and Animal Welfare of the Universitat Politècnica de València (UPV), following the Spanish Royal Decree 53/2013 on the protection of animals used for scientific purposes.

## 3. Results

### 3.1. CATA

Significant differences were observed (Table 3) among CATA data (*p* < 0.05) in the randomization test and pairwise comparison, suggesting differences among attributes and treatments regarding consumers’ assessments. The first column shows *p*-values associated with Cochran’s Q tests which compare products independently for each attribute. Differences were detected between the commercial group (considered in this study as an adequate fish quality) and the rest of the experimental diets, including the control.

A total of six attributes were significant, i.e., there were differences among the treatments assayed. The multiple pairwise comparisons test considering commercial fish as the “acceptable food” since it represents the product that consumers are usually used to, which is a requirement to be able to develop this test, demonstrated that significant differences were found between commercial seabass and the rest of the treatments studied regarding white color, toasted aspect, greyish color, sourness taste and faint flavor.

**Figure 1:**
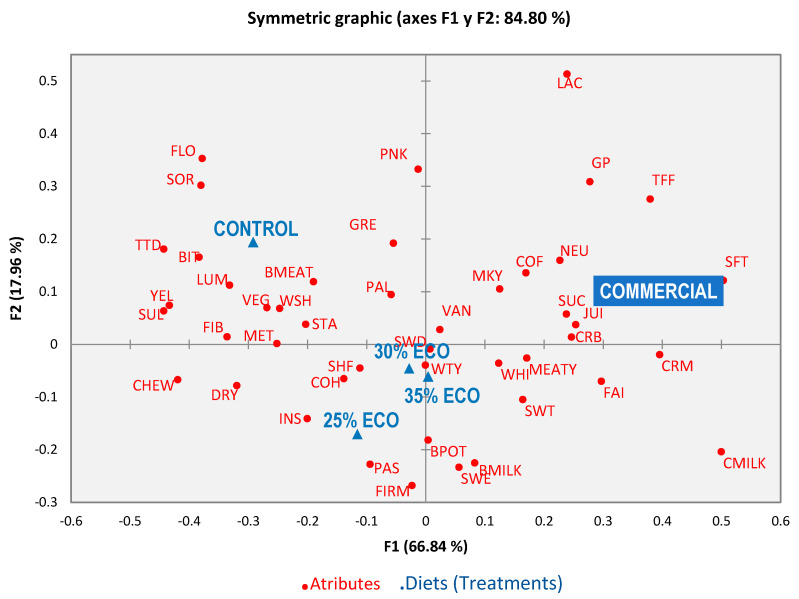
Biplot representation acquired by CATA for seabass fillets. Sweet flavor (SWE), boiled milk (BMILK), corn flour (COF), shellfish (SHF), seaweed (SWD), boiled meat (BMEAT), green plant (GP), neutral (NEU), wood shaving (WSH), vanilla (VAN), toffee (TFF), condensed milk (CMILK), boiled potato (BPOT), milky (MKY), lactic acid (LAC), watery (WTY), metallic (MET), starchy (STA), sweetness (SWT), meaty (MEATY), vegetable (VEG), faint (FAI), insipid (INS), sourness (SOR), bitter (BIT), sulphide (SUL), yellowish (YEL), white (WHI), toasted (TTD), pale (PAL), pink (PNK), greyish (GRE), cohesive (COH), succulent (SUC), creamy (CRM), lumpy (LUM), pasty (PAS), juicy (JUI), fibrous (FIB), dry (DRY), softness (SFT), crumbly (CRB), floury (FLO), chewy (CHW) and firm (FIR).

The comparison among experimental treatments showed that the toasted aspect was similar between the control and the 25% ECO but distinct from the rest of organic diets. The greyish color was different for the control and the 35% ECO. Regarding sour taste, the control only showed differences compared to the 25% ECO. Additionally, the faint flavor of the 35% ECO resulted in a distinct flavor compared to the control. Concerning texture, especially softness, again the 35% ECO was different from the control and the 25% ECO but was similar to the 30% ECO. Shellfish and seaweed odors did not show significant differences; however, their proportion means show that they were frequently ticked by consumers for the tested treatments. The independence between rows and columns was confirmed by a Chi-square test (*p* ≤ 0.05). This finding indicates that it is very likely that there are real differences in sensory profiles among treatments, including the commercial treatment. The table of the eigenvalues and the corresponding plot (Figure 1) allows for verifying the quality of the analysis developed.

The quality of the analysis was adequate (84.8% of the explained total inertia on the first two dimensions). According to the CATA map, the commercial group (cooked fillets) were relatively soft, creamy, juicy, crumbly, succulent, with a neutral odor and had a typical meaty flavor. For its part, the control diet resulted in a floury, lumpy texture, bitter flavor and a boiled meat odor. All organic diets were well related to a shellfish odor, but the 30% ECO and the 35% ECO also had a seaweed odor, a cohesive texture and watery flavor, while the 25% ECO resulted in a pasty texture, boiled potato odor and an insipid taste.

**Figure 2:**
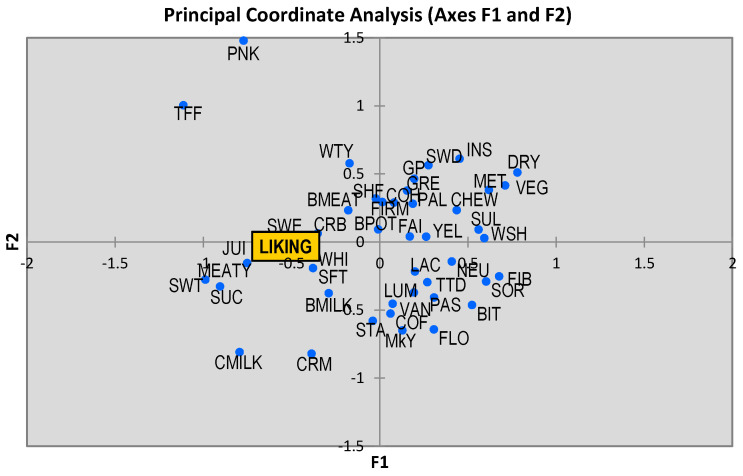
Bidimensional map from PCoA for seabass fillets. Sweet (SWE), boiled milk (BMILK), corn flour (COF), shellfish (SHF), seaweed (SWD), boiled meat (BMEAT), green plant (GP), neutral (NEU), wood shaving (WSH), vanilla (VAN), toffee (TFF), condensed milk (CMILK), boiled potato (BPOT), milky (MKY), lactic acid (LAC), watery (WTY), metallic (MET), starchy (STA), sweetness (SWT), meaty (MEATY), vegetable (VEG), faint (FAI), insipid (INS), sourness (SOR), bitter (BIT), sulphide (SUL), yellowish (YEL), white (WHI), toasted (TTD), pale (PAL), pink (PNK), greyish (GRE), cohesive (COH), succulent (SUC), creamy (CRM), lumpy (LUM), pasty (PAS), juicy (JUI), fibrous (FIB), dry (DRY), softness (SFT), crumbly (CRB), floury (FLO), chewy (CHW) and firm (FIR).

Tetrachoric correlations between attributes and biserial correlations, developed between a binary variable (sensory attributes) and a quantitative variable (liking) have been calculated and are visualized by principal coordinate analysis (PCOA) whose results are shown in a two-dimensional map (Figure 2). The screen plot indicates that the two first dimensions were sufficient to interpret relationships between attributes with accumulative variances of 25.91% and 35.66% to F1 and F2, respectively. Based on proximities among attributes, PCoA plot shows that “liking” was associated with a sweet odor, soft and crumbly texture, juicy, as well as with a typical meaty taste. According to this representation, these seem to be the attributes that Spanish consumers want to find in a fillet of seabass after cooking.

#### Penalty Analysis

Based on the CATA findings, a penalty analysis was used to identify both drivers of liking and opposing factors. Namely, those attributes that consumers consider as negative. When liking data is available, it can be related to the analysis. A first test is based on incongruence in which the attribute is missing in the assessed fish (treatments ECO and the control) but not in the commercial treatment, considered as a satisfactory product for the consumer. This allows the identification of the “must-have” attributes by building a summary table that indicates the frequencies with which P(No)|(Yes) and P(Yes)|(Yes) occurs for each attribute.

Table 4 shows the mean drops and their significance in liking between the two situations (present/absent) for each attribute that was relevant. In this sense, meaty flavor involved an increase of 1.21 liking points between tested products and the ideal product. The same condition was observed for white color, juicy texture and soft texture. All of them were considered as “must-have”. After that, a second analysis, similar to the previous one but based on incongruence in which the characteristic was missing in the adequate quality fish (commercial) but not in the tested diets (control and organic diets), allowed the identification of “nice to have” attributes when positive mean impacts were found or, conversely, “must not have” when impacts resulted negative. In this sense, the fibrous texture showed a standardized difference for a mean drop of −3.073 (*p* < 0.002); therefore, it was considered as a “must not have” attribute. Furthermore, this test was also useful to detect “does no harm” which are those attributes that were not selected in the previous categories and are considered as indifferent and with little relevance. The rest of the attributes considered in this study were located inside this group.

### 3.2. Projective Mapping

**Figure 3:**
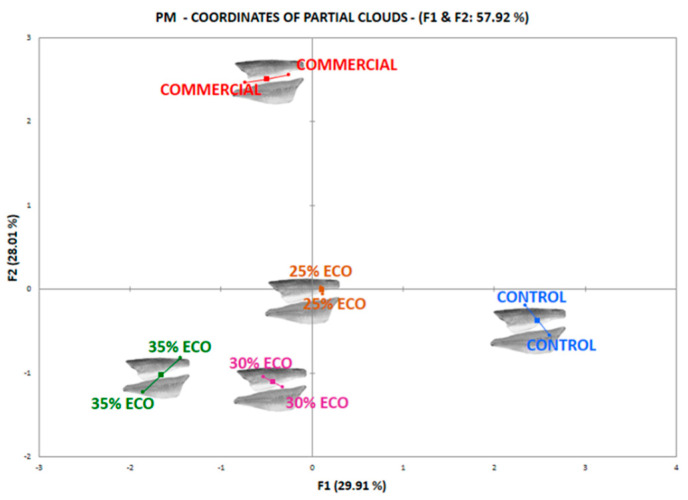
Projective map provided by multiple factorial analysis for the opinion of consumers about seabass fillets from experimental diets and commercial samples. COMMERCIAL: Fish for sale. CONTROL: Control diet and organic diets (ECO) with different levels of inclusion of fishmeal (25%, 30% and 35%. i.e., diets 25% ECO, 30% ECO and 35 ECO).

The first two dimensions of MFA accounted for approximately 57.92% of the variance of the experimental data. According to Figure 3, the F1 (29.91%) dimension collected the most considerable variation among diets, where the control contributed with 65.8%, while the 35% ECO showed 29.4%. The commercial treatment had the most important weight in F2 (28.01%) with 72.3%. Treatments 35% ECO and 30% ECO represented less than 26% whereas the 25% ECO remained very close to the center of the graphic. Although F3 does not appear in the graph, it should be noted that organic diets with amounts of ≥ 30% (30% ECO and 35% ECO) represented 64% of the variation in this axis. Consumers were able to separate commercial fish from experimental diets, they also discriminated between the control diet of the rest of the treatments with the organic feed addition, which made a small group among them.

### 3.3. Comparative Study between Methodology (CATA vs. PM)

**Figure 4:**
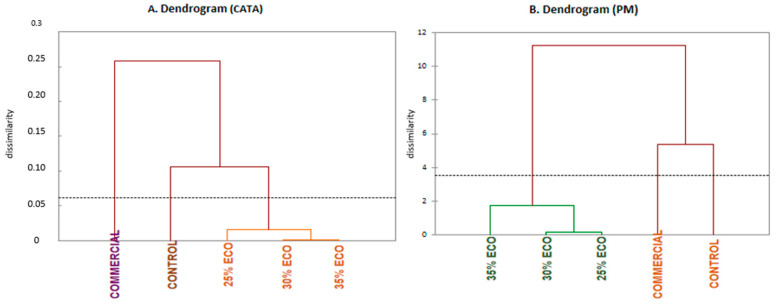
Dendrograms from the cluster provided by CATA (**A**) and PM (**B**) for seabass fillets. COMMERCIAL: Fish for sale. CONTROL: Control diet and organic diets (ECO) with different levels of inclusion of fishmeal (25%, 30% and 35%, i.e., diets 25 ECO, 30 ECO and 35 ECO).

As shown in Figure 4, dendrograms from HCA performed for both methods had a similar configuration and were effective to discriminate among organic diets (ECO), control and commercial, forming three distinct clusters. The fish fed with control diet results were very different from commercial fish as well as from those fed with organic diets (ECO), which remained close and conformed to a small group with different behavior depending on the method considered. CATA (Figure 4A) demonstrated that 25% ECO was different from the couple formed by 30% ECO and 35% ECO. In contrast, PM (Figure 4B) showed a small group, too, but 30% ECO and 25% ECO were similar while the distinct diet was 35% ECO. These results suggest that there were changes in the perception of organic diets among consumers. Maybe the method applied to discriminate can influence the choice of the consumer for one or the other diet (35% ECO or 25% ECO).

CATA and PM demonstrated significant and high compatibility with similar values between them (RV = 0.895 and *p* = 0.012). As a result, it can be regarded as an indicator of good agreement between the sample configuration.

**Figure 5:**
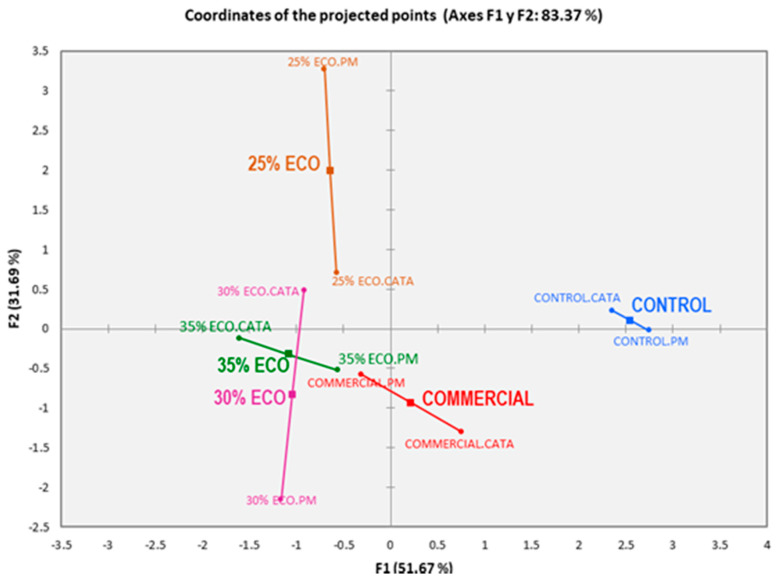
Comparative configurational map generated by Multifactor Analysis (MFA) on the individual configuration of Check-All-That-Apply (CATA) and projective mapping (PM). About the general position in the plot: Commercial: Fish for sale, Control: Fish fed without organic ingredients, and ECO: fish fed with organic diets with different levels of inclusion of fishmeal (25%, 30% and 35%, i.e., 25 ECO, 30 ECO and 35 ECO). Regarding CATA and PM, abbreviates after names indicate location according to each method.

Figure 5 shows the MFA plot for this comparative study, demonstrating that it represents 83.3% of the total variance of the study. Changes in consumers’ perception of sensory attributes for different organic diets, especially in 30% ECO and 35% ECO were appreciated. Both methods (PM and CATA) separated the control from the other diets; commercial has located close to 30% ECO and 35% ECO diets but away from 25% ECO. Regarding commercial, it seems that the criterion of consumers was reasonably consistent for fish from retail. Additionally, for organic diets with the inclusion of more than 30% fish meal, there seems to be an agreement since they were located very close to each other and formed a small group that included commercial. The criteria of both methods (CATA and PM) coincided satisfactorily for the 35% ECO diet while in 30% ECO a significant discrepancy was detected between sensory methods, almost as large as those detected in 25% ECO, in which the PM’s criterion was moved close to the ends in the plot. This last diet was also located separately from all the others and highlights its proximity with treatments 30% ECO according to CATA. As mentioned before, this could be due to differences in the perception of consumers regarding fish attributes.

From a practical point of view, the quality of seabass obtained with organic feeding and with reduced fish meal inclusion rates was different among all the treatments assayed. Thus, sensory attributes showed by organic diets ECO were different from those for the control and commercial.

## 4. Discussion

### 4.1. CATA

The current study aimed at investigating the opinion of consumers about the quality of seabass produced with organic feeding and with reduced fish meal rates. Significant differences (*p* ≤ 0.05) were found between commercial and all experimental diets assayed (ECO and control). Unlike seabass from experimental diets, fish for sale (commercial) represents real food that is currently offered on the market. In essence, traditional aquaculture seabass sold on the retail market already has recognition (product loyalty) from customers who are also already familiar with its sensory attributes due to its frequent purchase or consumption [7]. Results in this test could be due to the type of farming production system, since fish for sale (commercial) were raised in the open sea (cages), while experimental fish were cultured in a closed system where a permanent recirculation of seawater was used to supply an adequate condition of the water for the fish. This process (recirculation) modifies original water characteristics, and this could affect sensory attributes of fish fillets in experimental treatments. However, among organic diets and the control, significant differences could be attributed mainly to the composition of diets because all experimental fish were produced in the same condition (recirculation closed system).

A sensorial characterization was carried out by penalty analysis to identify both drivers of liking and opposing factors. The meaty flavor, white color, juiciness, and soft texture were desirable attributes while the fibrous texture was the main negative aspect for seabass fillets tested. An advantage offered by a penalty analysis is that it allows the visualization of the relationships among attributes that help to choose characteristics to be considered together in the context of improving a food product [30]. PCoA plot obtained from CATA data confirms these findings and shows clearly those attributes associate with “liking” as happened in a previous study [29].

### 4.2. Projective Mapping

According to the bibliography in this area [11,12,13], developing a PM technique is requiring a sample size with a minimum number of eight and a maximum number that could be variable depending upon the nature of the products assayed. In this sense, this work shows a methodological limitation since a total of five products were used (less than six) and this could affect both the effectiveness of the method and the reliability of the results obtained. For this reason, it was necessary to carry out a comparative study with CATA to establish the similarity between results.

The preference for the control, commercial and organic diets with more than 30% demonstrated to be different once an MFA was carried out, due to the sensitivity of this test, to detect differences among assessors [24]. The affective approach to projective mapping reveals consumers’ drivers of liking and choice from a holistic perspective just as they would do when choosing in real-life situations according to their preferences [32]. Once again, consumers identified commercial fish as different and demonstrated a moderate ability to discriminate among diets with different organic feed inclusion (%), separating diets with more than 30% from a diet with a lower addition. The idea of mapping is to first collect, through the positioning of the stimuli, the first and the second reason why the stimuli are perceived as different for each subject. Then, to summarize all these reasons over all subjects [8].

This finding is key for the aquaculture industry, especially for farmers since taking into account production cost for farmed seabass, a reduction from 35 to 30% represents an important impact over the final price that consumers have to pay for these organic products, and it is well knowing that the price is a very important driver of fish purchase [33].

### 4.3. Comparative Study between Methodology (CATA vs. PM)

Results of HCA showed differences in dendrograms of each sensory methodology applied. Perhaps this finding is due to different cognitive strategies encouraged in the sensory characterization tasks, leading to varying forms of activation of neural activity, recognition and recall during the tests [32]. In this sense, projective mapping is an intuitive method characterized by its holistic nature and based on the global perception of consumers, which identifies only the main attributes that distinguish samples. Conversely, CATA questions are based on recognition activities, the information is already available, and the answers are chosen from a list containing different alternatives [34]. The subjects involved in the CATA experiment are generally not trained. Therefore, they may differ in the interpretation of the attributes or may have different perceptions of the products. The segmentation of these subjects is of paramount interest and this issue has not sufficiently been addressed in the literature [35].

Following the results provided by MFA for CATA and PM, 30% ECO would be an adequate formulation for seabass feed since it would allow achieving a fillet more alike to the commercial product than to the control’s diet; it could be differentiated, too, from diets with other substitution amounts (%). From a marketing point of view, the most relevant topic is to tackle the perception towards the quality of farmed fish and its feed, which is, as a matter of fact, the guarantee of the organoleptic and nutritional quality of aquaculture products [36,37].

The aquafeed industry is facing a lack of availability of fish meal and oil. The need for economic and environmental sustainability has increased the use of plant-derived oils and for that, it is making efforts to reduce the dietary inclusion of marine ingredients, searching alternative dietary proteins and lipid sources, and formulating more efficient eco-friendly feeds [38]. In addition to the above, product quality and its service characteristics would naturally pave the way for a consumer, bringing about his satisfaction and ultimately, gaining his loyalty [39]. However, it is known that consumers with higher objective knowledge about fish and a higher level of education are more ready to agree with scientific evidence and consequently more likely to make better and reasoned fish choices. Thus, the design of effective information strategies about farmed fish and its production aspects might help to increase its image and acceptance [40]. In this work, seabass was fed with organic diets, with more than 30% replaced with organic vegetables, which closely resembles commercial fish, and this finding would be an important aspect to disclose as it could modify the purchasing habits of the consumers toward sustainable, “environmentally friendly” products with a strongly positive impact on product evaluation and ultimately purchasing decisions [37].

## 5. Conclusions

The sensory methods applied in this study demonstrated that assessors were capable of discriminating among diets, indicating that all diets tested were different from commercial samples. CATA provided a sensory profile for each treatment assayed, especially for organic diets. This analysis was able to effectively recognize the characteristics found in the cooked meat of fillets from farmed seabass, establishing sensory attributes considered as “must-have” according to consumer´s criteria, chiefly white color, soft texture, tasty flavor and juiciness. Meanwhile, despite limitations due to the size of the sample used, projective mapping satisfactorily separated commercial fish from those belonging to the control and organic diets. In the last group, it identified the diet with a minor replacement (25%) as different. In summary, both sensory methods, despite their distinct conceptual approaches, were able to discriminate in a similar way all treatments assayed in this study. More relevant is the fact that fishes reared in tanks with organic feed at 30–35% were very close in consumer liking to those reared in open sea cages.

The information obtained in this study provides interesting insights into the aquaculture sector, especially for Mediterranean fish farmers, since the overall analysis (MFA) showed, based on the sensory perception, that seabass from organic diets (>30%) had in consumers a quality similar to that of commercial fish. Hence, a key policy recommendation, based on this study inside the “blue revolution” [5] of the aquaculture industry, would be to promote the benefits of organic farmed fish consumption for sustainable activity, and could contribute to eradicating consumer beliefs about the poor sensory quality of farmed fish.

The result of this research demonstrated that check-all-that-apply, in combination with projective mapping, might be a useful tool for the new product development process, especially in aquaculture. This study represents an opportunity to show that sensometrics offer the aquaculture industry an accurate discriminating approach that takes into account the consumers’ point of view.

## Figures and Tables

**Table 1:** General information for the panel of consumers (*n* = 100) that was used in this study.

Socio-Demographic Data			Purchase Data		
Variable	Categories	Frequency (%)	Variable	Categories	Frequency (%)
Gender	Male	60	Buyer of fresh whole seabass	NO	59
	Female	40		YES	41
Nationality	National	70	Place of purchase of fish	Hypermarket	5
	Foreigner	30		Food market	7
Ages	18–24	27	Purchase frequency of fish	Supermarket	88
	25–34	34		>once a week	2
	35–44	16		once a week	32
	45–54	12		<once a week	39
	55–65	11		<once a month	27
Economic status	Salaried	57	Way of sale (only fresh seabass)	Whole fish	39
	Self-employed	10		Eviscerated fish	61
	Unemployed	4			
	Student	29			
Home income (per month)	<900 EUR	16	Size (only fresh seabass)	Big (600–1000 g)	24
	901–1800 EUR	23		Medium (400–600 g)	68
	1801–3000 EUR	37		Small (200–400 g)	7
	>3000 EUR	22			
Children at home	NO	82	Origin (only fresh seabass)	Imported	59
	YES	18		Local	41

**Table 2:** Sensory terms compressed in the CATA ballot for ecologic seabass.

No.	CODE	I. Odor	No.	CODE	II. Meat color
1	SWE	Sweet smell	16	YEL	Yellowish
2	BMILK	Boiled milk	17	WHI	White
3	COF	Corn flour	18	TTD	Toasted
4	SHF	Shellfish	19	PAL	Pale
5	SWD	Seaweed	20	PNK	Pink
6	BMEAT	Boiled meat	21	GRE	Greyish
7	GP	Green plant			
8	NEU	Neutral	**No.**	**CODE**	**IV. Texture**
9	WSH	Wood shaving	33	COH	Cohesive
10	VAN	Vanilla	34	SUC	Succulent
11	TFF	Toffee	35	CRM	Creamy
12	CMILK	Condensed milk	36	LUM	Lumpy
13	BPOT	Boiled potato	37	PAS	Pasty
14	MKY	Milky	38	JUI	Juicy
15	LAC	Lactic acid	39	FIB	Fibrous
			40	DRY	Dry
**No.**	**CODE**	**III. Flavor/Taste**	41	SFT	Soft
22	WTY	Watery	42	CRB	Crumbly
23	MET	Metallic	43	FLO	Floury
24	STA	Starchy	44	CHW	Chewy
25	SWT	Sweet	45	FIR	Firm
26	MEATY	Meaty			
27	VEG	Vegetable			
28	FAI	Faint			
29	INS	Insipid			
30	SOR	Sour			
31	BIT	Bitter			
32	SUL	Sulphuric			

**Table 3:** Q test and multiple pairwise comparisons use the critical differences for products (control & ecological diets (ECO)) and attributed tested.

Attributes	CODE	*p*-Value	TESTED DIETS (Proportion Means)			
			CONTROL	25% ECO	30% ECO	35% ECO
Shellfish odor	SHF	0.830	0.450 a	0.470 a	0.420 a	0.430 a
Seaweed odor	SWD	0.463	0.300 a	0.310 a	0.350 a	0.270 a
White color	WHI	0.034 *	0.570 a	0.720 a	0.630 a	0.720 a
Toasted color	TTD	0.003 *	0.260 b	0.160 ab	0.110 a	0.110 a
Greyish color	GRE	0.004 *	0.160 b	0.090 ab	0.180 b	0.050 a
Sour taste	SOR	0.046 *	0.150 b	0.040 a	0.090 ab	0.090 ab
Faint flavor	FAI	0.030 *	0.110 a	0.180 ab	0.220 ab	0.250 b
Soft texture	SFT	0.017 *	0.150 a	0.140 a	0.200 ab	0.290 b

* Different letters denote significant differences among treatments (*p* < 0.05).

**Table 4:** Summarized comparison for frequency of occurrence to identify the must-have attributes in seabass fillets tested.

Variable	Level	Frequency	%	Sum (LIKING)	Mean (LIKING)	Mean Drops	Standardized Difference	*p*-Value	Significant	Penalties
NEU	P(N)I(Y)	80	20	248	3.10	0.35	1.476	0.304	No	
	P(Y)I(Y)	24	6	83	3.45					0.232
WHI	P(N)I(Y)	100	25	293	2.93	0.41	3.410	0.001	Yes	
	P(Y)I(Y)	228	57	763	3.34					0.248
MEATY	P(N)I(Y)	102	25.5	290	2.84	1.21	8.092	<0.0001	Yes	
	P(Y)I(Y)	70	17.5	284	4.05					0.990
FAI	P(N)I(Y)	86	21.5	254	2.95	0.13	0.644	0.796	No	
	P(Y)I(Y)	34	8.5	105	3.08					−0.166
JUI	P(N)I(Y)	100	25	281	2.81	0.99	5.821	<0.0001	Yes	
	P(Y)I(Y)	52	13	198	3.80					0.653
SFT	P(N)I(Y)	144	36	446	3.09	0.51	3.097	0.006	Yes	
	P(Y)I(Y)	52	13	188	3.61					0.431
CRB	P(N)I(Y)	112	28	354	3.16	0.36	2.310	0.056	No	
	P(Y)I(Y)	68	17.	240	3.52					0.349

Neutral Odor (NEU). White color (WHI). Meaty flavor/taste (MEATY). Faint flavor (FAI). Softness texture (SFT) and crumbly texture (CRB).
